# Response to rituximab in B-CLL patients is adversely impacted by frequency of IL-10 competent B cells and FcγRIIIa polymorphism. A study of FCGCLL/WM and GOELAMS groups

**DOI:** 10.1038/bcj.2015.115

**Published:** 2016-01-22

**Authors:** A-L Gagez, E Tuaillon, R Cezar, C Dartigeas, B Mahé, R Letestu, H Maisonneuve, V Gouilleux-Gruart, K Bollore, E Ferrant, T Aurran, P Feugier, S Leprêtre, G Cartron

**Affiliations:** 1 CNRS UMR 5235, Université de Montpellier, Montpellier, France; 2 INSERM U1058, Université de Montpellier, Montpellier, France; 3 Département de Bactériologie-Virologie CHRU de Montpellier, Montpellier, France; 4 Département d'Hématologie Clinique, CHRU de Tours, Tours, France; 5 Département d'Hématologie Clinique, CHRU Nantes, Nantes, France; 6 APHP, GHUPSSD, Hôpital Avicenne, Service d'hématologie biologique, Bobigny, France; 7 Service de médecine onco-hématologie, CHD La Roche sur Yon, La Roche sur Yon, France; 8 CNRS UMR 7292, Université François Rabelais, CHRU de Tours, France; 9 Département d'Hématologie Clinique, CHRU Dijon, Dijon, France; 10 Centre Paoli Calmette, Marseille, France; 11 Département d'Hématologie Clinique, CHRU Nancy, Nancy, France; 12 Centre Henri Becquerel, Rouen, France; 13 Département d'Hématologie Clinique, CHRU de Montpellier, Montpellier, France

Rituximab (MabThera, Rituxan) *in vivo* mechanisms of action remain incompletely understood and could differ depending on the subtype of B-lymphoproliferative disorders. Rituximab has been shown to induce apoptosis, complement-mediated lysis, antibody-dependent cellular cytotoxicity (ADCC) and antibody-dependent phagocytosis (ADPC) *in vitro* and there is some evidence pointing towards an involvement of these mechanisms *in vivo*. Factors affecting rituximab response have been previously described including histology, tumor burden and rituximab pharmacokinetics.^1^ We previously observed that FcγRIIIa-158 V/F polymorphism also impacts on clinical response. Because this polymorphism affects human IgG1 affinity for FcγRIIIa expressed on both natural killer cells and macrophages, we postulated that ADCC was an important mechanism of rituximab activity in follicular lymphoma.^2^ New anti-CD20 antibodies, with higher affinity for FcγRIIIa and ADCC such as obinutuzumab, have been therefore developed and are currently in clinical development.

Regulatory B cells have been identified in human and mice as a subset of B lymphocytes competent to secrete interleukin-10 (IL-10). These cells, also named B10 in mice, are characterized by their ability to modulate inflammation, autoimmunity and adaptive and innate immune response through the production of IL-10.^3, 4, 5^ In mouse model, B10 cells would inhibit lymphoma cell clearance induced by anti-CD20 monoclonal antibodies (mAbs) through the regulation of monocyte Fc-mediated functions. Recently, it has been demonstrated that clonal chronic lymphocytic leukemia (CLL) cells displayed such IL-10 competence and immunosuppressive functions.^6^ We therefore hypothesized that IL-10-competent B-CLL cells could influence the clinical efficacy of rituximab in CLL patients.

A prospective, randomized phase II study (NCT01370772) including 140 patients was conducted between June 2012 and January 2013 in France. Treatment-naive patients (aged 18–66 years) diagnosed with Binet stage C or active Binet stage A or B CLL were enrolled.^7^ Inclusion criteria are described in the supplementary Methods available on the *Blood Cancer Journal* website. In the experimental arm, standard fludarabine-cyclophosphamide-rituximab (FCR) courses (six 28-day courses of rituximab: 375 mg/m^2^ D1 C1 and 500 mg/m^2^ D1 C2–C6; fludarabine: 40 mg/m^2^/d D2–4, cyclophosphamide: 250 mg/m^2^/d D2–4) were preceded by a prephase of rituximab: 500 mg on D0 and 2000 mg on D1, D8 and D15. Immuno-chemotherapy was planned to begin at D22. Primary end point results have been previously published.^8^

We considered that lymphocyte depletion after rituximab monotherapy assessed at D22 was a surrogate marker of *in vivo* rituximab activity, allowing thus to analyze influence of IL-10-competent B-CLL cells on *in vivo* rituximab efficacy, in the 68 patients included in the experimental arm. Median lymphocyte count before the four doses of rituximab (D0) was 91.13 g/l (range: 3.74–497.40) and was 2.60 g/l (range: 0.14–189.40) at the end of rituximab prephase (D22). Thus the median lymphocyte depletion after rituximab prephase (D22) was 95.1% (range: −77.0 to +99.9), among them 66% obtained more than 90% depletion. Patients' characteristic and their distribution according to 90% lymphodepletion are presented in Table 1. No significant correlation was found between 90% lymphodepletion and clinical (age, sex, Binet stage, Eastern Cooperative Oncology Group Performance Status) and biological (*IGHV* mutation, cytogenetic abnormalities and β2-microglobulin) parameters. A subset of IL-10-competent B-CLL cells was detected in all patients tested (*n*=47, median: 3.06% of CLL cells, range: 0.12–29.55). High frequency of IL-10-competent cells among B-CLL cells was associated with a high IL-10 plasma level (Figure 1a, *r*=0.40, *P*=0.017) whereas the IL-10 plasma level did not correlate with the percentage of IL-10 negative B-CLL cells (data not shown). The frequency of IL-10-competent B-cells did not differ according to the characteristics of the patients, and was not significantly different in CLL cases with unmutated vs mutated *IGVH* (median: 6.29%, range: 0.12–15.83 vs median: 1.85%, range: 0.23–20.81, respectively). In addition, IL-10-competent B-cell frequency was not associated with cytogenetic alterations (del11q, del13q, trisomy 12). Such results were in agreement with a previous report.^6^ Univariate analysis showed that the frequency of IL-10-competent B-CLL cells adversely impacted on 90% lymphodepletion observed after rituximab prephase (D22) (Figure 1b, *P*=0.004). In addition, IL-10-competent B-cell frequency was also found to correlate with clinical response assessed 3 months after immuno-chemotherapy by FCR (complete response (CR) vs no-CR, *P*=0.04). No correlation was found between lymphodepletion after rituximab prephase and response to immuno-chemotherapy (CR vs no-CR, *P*=0.96). Thus, these results suggested that all CLL patients have a subpopulation of IL-10-competent B-CLL cells, which represent a variable percentage of B-CLL cells and exert a clinically significant inhibitory effect on *in vivo* rituximab activity, probably through IL-10 secretion. Because FcγRIIIa-158V/F polymorphism correlates with *in vivo* efficacy of rituximab in follicular lymphoma, we determined FcγRIIIa-158V/F polymorphism in our cohort of patients. FcγRIIIa*-*158V/F polymorphism was significantly associated with 90% lymphodepletion (*P*=0.028) and a normal lymphocyte count (<5g/l) (*P*=0.028) at D22. This was also found for FcγRIIIa*-*158V carriers (*P*=0.014) (Figure 1c). FcγRIIIa-158V/F polymorphism failed however to correlate with clinical response 3 months after immuno-chemotherapy by FCR. Thus, these results suggest that FcγRIIIa-mediated immune functions play a critical role in clinical rituximab activity, but the impact of FcγRIIIa-158V/F polymorphism on immuno-chemotherapy response could be masked either by the high activity of immuno-chemotherapy or by direct inhibition of immune effector cells by chemotherapy. This is the first report demonstrating the influence of FcγRIIIa*-*158V/F polymorphism in CLL patients. This is probably related to the fact that it is also the first study analyzing such influence in monotherapy context. These results could also explain clinical superiority of the first Fc-glycoengineering anti-CD20 antibody exhibiting high affinity for FcγRIIIa (obinutuzumab), compared with rituximab reported in CLL patients.^9^

Logistic regression analyses showed that only frequency of IL-10-competent B-CLL cells and FcγRIIIa-158V/F polymorphism was associated with 90% lymphodepletion after rituximab prephase (odds ratio (OR)=0.83; 95% confidence interval (CI): 0.72–0.93; *P*=0.002 and OR=4.95; 95% CI: 1.07–27.48; *P*=0.043, respectively). The receiver operating characteristic curve using IL-10-competent B-cells frequencies and FcγRIIIa-158V/F polymorphism showed a highly discriminative power (area under the curve=0.855; 95% CI: 0.732–0.978), leading to predict patients who will have more than 90% of lymphodepletion after rituximab prephase (Figure 1d).

The experimental model suggested that IL-10-producing B cells would inhibit macrophage-mediated lymphoma depletion induced by anti-CD20 mAbs.^10^ Human macrophages express FcγRIIIa which mediates ADCC induced by rituximab whereas FcγRIIa, also expressed by macrophages, mediates ADPC. Data of this preliminary study indicate that IL-10-competent B-CLL cells frequency correlates with the ability of rituximab to induce B-CLL cells depletion. This effect seems especially important since IL-10-competent B-CLL cells frequency influenced also clinical response after immuno-chemotherapy. The depletion of B-CLL cells was also independently influenced by FcγRIIIa-158V/F polymorphism with a better response for FcγRIIIa-158V carriers, which have a better affinity for human IgG1. The influence of FcγRIIIa-158V/F polymorphism was however not found after immuno-chemotherapy, indicating that chemotherapy probably negatively impacts on FcγRIIIa-expressing cells. In conclusion, all these data lead us to hypothesize that IL-10-competent B-CLL cells negatively regulate rituximab-mediated ADCC by macrophages in CLL patients, this effect being modulated by FcγRIIIa-158V/F polymorphism. Strategies targeting IL-10-mediated inhibitory effects should be considered to improve rituximab efficacy.

## Figures and Tables

**Figure 1:**
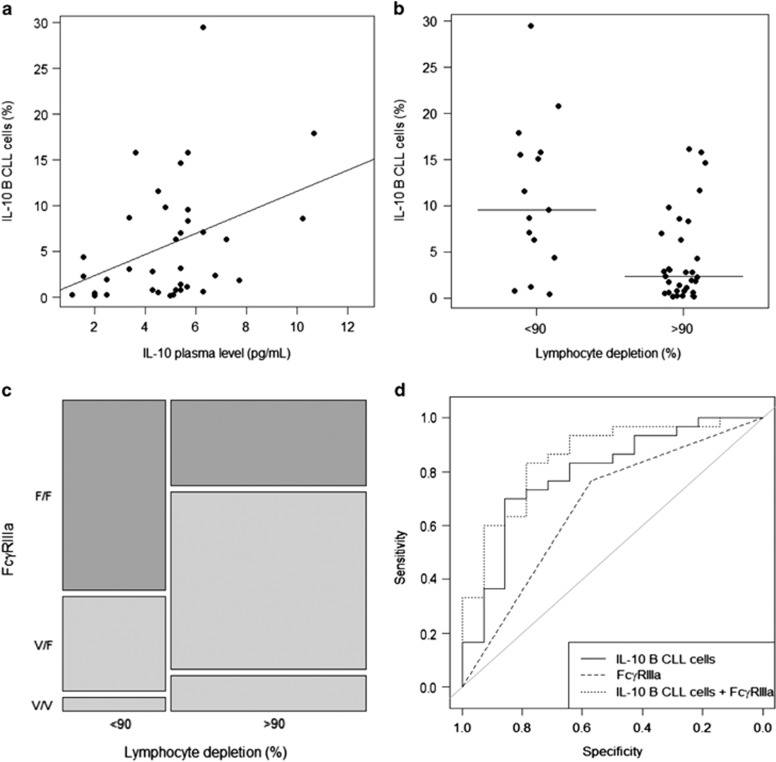
Effects of IL-10 B-CLL cells frequency and FcγRIIIa-158V/F polymorphism on lymphocyte depletion induced by rituximab in CLL patients. (**a**) IL-10-competent B-CLL cells frequency correlated with IL-10 plasma level at D0 (*P*=0.017). (**b**) Lymphocyte depletion observed after prephase of rituximab alone (D22) was statistically influenced by IL-10-competent B-CLL cells frequency at D0 (*P*=0.004). (**c**) Lymphocyte depletion between D0 and D22 was statistically higher for FcγRIIIa-158V carriers patients (*P*=0.014). (**d**) Receiver operating curve (ROC) generated using logistic regression for IL-10-competent B-CLL cells alone (area under the curve (AUC)=0.763), FcγRIIIa-158V/F polymorphism V carriers vs F/F (AUC=0.675) and combination of IL-10-competent B-CLL cells and FcγRIIIa-158V/F polymorphism V carriers vs F/F (AUC=0.855).

**Table 1:** Parameters influencing lymphocyte depletion induced by rituximab monotherapy in 68 CLL patients

	*Lymphodepletion >90% (*n=*44)*		*Lymphodepletion ⩽90% (*n=*23)*		*Univariate analysis*			*Multivariate analysis*	
	N *(%)*	*Median (IQR)*	N *(%)*	*Median (IQR)*	*OR (95% IC)*	P*-value*	*AUC (95% CI)*	*OR (95% CI)*	P*-value*
Age (years)	—	55.72 (51.31–58.12)	—	53.99 (52.07–57.41)	—	0.792	—		
Men	31 (70.45)	—	18 (78.26)	—	0.68 (0.19–2.16)	0.693	—	—	—
Binet stage AB	36 (81.82)	—	16 (69.57)	—	0.51 (0.15–1.73)	0.404	—	—	—
ECOG 0	31 (70.45)	—	5 (21.74)	—	1.50 (0.41–6.29)	0.572	—	—	—
*IGHV* unmutated	25 (56.82)	—	16/22 (72.73)	—	0.50 (0.15–1.50)	0.324	—	—	—

*Cytogenetic abnormalities*									
Del(13q)	18/35 (51.43)	—	8/18 (44.44)	—	1.31 (0.41–4.29)	0.848	—	—	—
** **Del(11q)	7/42 (16.67)	—	6/23 (26.09)	—	0.57 (0.16–2.07)	0.560	—	—	—
Trisomy 12	2/33 (6.06)	—	2/14 (14.29)	—	0.40 (0.03–6.04)	0.572	—	—	—
β2-microglobulin (mg/l)	39 (88.64)	2.90 (2.33–3.66)	22 (95.65)	2.76 (2.33–4.29)	—	0.857	—	—	—
CD38+ (%)	34 (77.27)	2.00 (0.00–23.00)	17 (73,91)	10.50 (1.75–26.00)	—	0.133		—	—
IL-10-competent cells (%)	32 (72.73)	2.30 (0.68–6.47)	15 (65.22)	9.51 (5.35–15.70)	—	0.004	0.763 (0.604–0.921)	0.83 (0.72–0.93)	0.002
*FCGR3A*					—	0.028	—	—	—
V/V	5 (11.91)	—	1 (4.55)	—					
V/F	25 (59.52)	—	7 (31.82)	—					
F/F	12 (28.57)	—	14 (63.63)	—					
*FCGR3A* V carrier	30 (71.43)	—	8 (36.36)	—	4.23 (1.43–13.42)	0.014	0.675 (0.551–0.799)	4.95 (1.07–27.48)	0.043

Abbreviations: AUC, area under the curve; ECOG, Eastern Cooperative Oncology Group Performance Status; IGHV, immunoglobin heavy-chain; OR, odds ratio; 95% CI, 95% of confidence interval.

Patients with less than 90% of lymphocyte count inhibition are used as the OR reference group.
