# Supplementary material for: Response to rituximab in B-CLL patients is adversely impacted by frequency of IL-10 competent B cells and FcγRIIIa polymorphism. A study of FCGCLL/WM and GOELAMS groups
Source: Blood Cancer J. 2016 Jan 22;6(1):e389–. doi: 10.1038/bcj.2015.115 (PMC4742632; doi:10.1038/bcj.2015.115)
Supplement: Supplementary Information [file bcj2015115x1.pdf]

Supplemental information.

Response to rituximab in B-CLL patients is adversely impacted by frequency of IL-10 competent B cells and FcγRIIIa polymorphism. A study of FCGCLL/WM and GOELAMS groups.

Anne-Laure Gagez,[1] Edouard Tuaillon,[2,3] Renaud Cezar,[3] Caroline Dartigeas,[4] Béatrice Mahé,[5] Rémi Letestu,[6] Hervé Maisonneuve,[7] Valérie Gouilleux-Gruart,[8] Karine Bollore,[2] Emmanuelle Ferrant,[9] Thérèse Aurran,[10] Pierre Feugier,[11] Stéphane Leprêtre,[12] Guillaume Cartron[1,13]

Affiliations

[1]CNRS UMR 5235, Université de Montpellier, Montpellier, France ; [2]INSERM U1058, Université de Montpellier 1, Montpellier, France ; [3]Département de Bactériologie-Virologie CHRU de Montpellier, Montpellier, France ; [4]Département d'Hématologie Clinique, CHRU de Tours, Tours, France ; [5]Département d'Hématologie Clinique, CHRU Nantes, Nantes, France ; [6]APHP, GHUPSSD, Hôpital Avicenne, Service d'hématologie biologique, Bobigny, France ; [7]Service de médecine onco-hématologie, CHD La Roche sur Yon, La Roche sur Yon, France ; [8]CNRS UMR 7292, Université François Rabelais, CHRU de Tours, France ; [9]Département d'Hématologie Clinique, CHRU Dijon, Dijon, France ; [10]Centre Paoli Calmette, Marseille, France ; [11]Département d'Hématologie Clinique, CHRU Nancy, Nancy, France ; [12]Centre Henri Becquerel, Rouen, France ; [13]Département d'Hématologie Clinique, CHRU de Montpellier, Montpellier, France.

Supplemental Methods.

CLL2010FMP protocol

Patients

A prospective, randomized, open-label, phase II study (NCT01370772) including 140 patients was conducted in 59 French centers between June 2012 to January 2013 (CLL-FMP 2010). Treatment-naïve patients (aged 18-66 years) diagnosed with immunophenotypically confirmed chronic lymphocytic leukemia according to IWCLL 2008 criteria with Binet stage C or with confirmed active disease in Binet stage A or B, were enrolled.[1] Additional inclusion criterion were the absence of 17p deletion, assessed by FISH (<10% positive nuclei). Each patient provided a written informed consent before enrolment.

Randomization

Patients were stratified according to *IGVH* mutational status FISH analysis (11q deletion or not) and were randomly assigned in one-to-one ratio to receive either standard dose of chemoimmunotherapy FCR (fludarabine, cyclophosphamide, and rituximab) for arm A or Dense-FCR with an intensified rituximab prephase before the standard treatment FCR for arm B.

Treatment

Standard treatment FCR arm consisted of six 28-day courses of rituximab (375 mg/m2 for the first course, D1 and 500 mg/m^2 for the others) fludarabine (40 mg/m^2/d D2-4), cyclophosphamide (250 mg/m^2/d D2-4). For the experimental arm, a FCR treatment was preceded by a prephase of rituximab composed of four successive infusions, distributed as follows: 500 mg on D0, and 2000 mg on D1, D8 and D15. Course 1 started at D22 for patients and subsequent courses every 28 days.

Objective

The main objective of this study was to increase complete response rate with undetectable minimal residual disease three months after treatment and was previously published.[2]

IL-10 competent B-CLL cells identification

IL-10 competent B-CLL cells counts were determined by flow cytometry analysis of IL-10 production. Peripheral blood mononuclear cells (PBMCs) were purified from peripheral blood samples of 68 arm B patients with CLL using Ficoll-Hypaque density gradients (Eurobio, Courtaboeuf, France).[3] PBMCs were resuspended (9 x 10^6 cells/mL) in medium (RPMI 1640 media (Biotech GmbH, Aidenbach, Germany) containing 10% fetal calf serum (Eurobio, Courtaboeuf, France), 2 mM L-glutamine (Eurobio, Courtaboeuf, France), 100 U/mL penicillin, 100 µg/mL streptomycin, and 2.5 µg/mL amphotericin (all antibiotics from Tebu-bio, Le Perray-en-Yvelines, France). Clonal activation of lymphocytes B (LB) were stimulated with CpG (ODN 2006, 10 µg/mL; InvivoGen, San Diego, USA), CD40L (50 ng/mL; R&D Systems, Minneapolis, MN, USA) and anti-polyHistidine (500 ng/mL; R&D Systems, Minneapolis, MN, USA) for 48 h at 37°C in a 5% CO_2–95% air humidified atmosphere. PMA (50 ng/mL; Sigma-Aldrich, Saint Louis, MO, USA) and ionomycin (1 µg/mL; Sigma-Aldrich, Saint Louis, MO, USA) were added on cells to stimulate IL-10 production. After 4 h at 37°C in a 5% CO_2–95% air humidified atmosphere, brefeldin A (1 X solution/mL; BioLegend, San Diego, CA, USA) blocked IL-10 secretion to determine B10pro + B10 cell population.[3-5] Antihuman antibody included: CD19 BV421 (HIB 19), CD69 PE/Cy7 (FN 50), CD38 APC (HIT 2), IL-10 PE (JES3-9D7) from BioLegend (San Diego, CA, USA), and CD45 KO (J.33) and CD5 FITC (BL1a) from Beckman Coulter (Brea, CA, USA). Clonal CLL cells were identified as CD19+ CD5+ CD20int lymphocytes. Analyses were performed on CyAn™ ADP flow cytometer (Beckman Coulter, Brea, CA, USA).

IL-10 assay

Plasma IL-10 level was measured using IL-10 Magnetic Luminex® Screening Assay and a Luminex dual laser, according to the instructions of the manufacturer (R&D Systems, Minneapolis, USA). Plasma of the 68 patients was incubated with superparamagnetic microparticles coated with IL-10 antibody

during 2h at room temperature, using 1:2 plasma dilutions. Plasmatic IL-10 was quantified with a combination of IL-10 biotinylated detection antibody and Phycoerythrin (PE)-conjugated streptavidin.

***FCGR3A* genotyping**

Single-step multiplex allele-specific PCR assays were performed as described by Dall'Ozzo et al. with minor modifications.[6] The 25 µL reaction mixture contained 10ng of genomic DNA, 400 nM of forward primer (5'-TCCAAA AGCCACACTCAAAGTC-3'), 400nM of reverse V allele primer (5'-AGACACATTTTTACTCCCATC -3') and 200nM reverse F allele primer (5'-GCGGGCAGGGCGGCGGGGGCGGGGCCGGTGATGTTCACAGTCTCTGATCACACATTTTTACTCCCATA-3'), 400 µM of each dNTP, 2mM $MgCl_2$ and 0.5U of Taq DNA polymerase in its buffer (Promega, Madison, USA). PCR conditions consisted in 3.5 min at 95°C followed by 35 cycles, each consisting in 95°C for 20 sec, 56°C for 20 sec, 72°C for 30 sec. After amplification PCR products (137bp fragment for F allele and 81bp for V allele) were resolved using 8% acrylamide gel (Invitrogen, Carlsbad, USA) and visualized after ethidium bromide staining.

Statistical analysis

Distributions of data were tested with the Shapiro-Wilk test. X^2 or Fisher test was used for categorical data. For numerical data, comparisons of medians were performed using Student T or Mann-Whitney test. All variables with a p value of <0.10 in univariate analysis were used for construction of Receiver Operator Curves (ROC) to determine the threshold associated with the best sensitivity and specificity, if necessary, and were included in an intermediate model. The final model variables were determined by backward selection using Student T test ($p<0.05$ as significant model). All statistical analyses were performed at the conventional two-tailed α level of 0.05 using R software version 3.0.2.[7]

Participating centers

CHU Amiens: Dr B. Royer; CHU Angers: Dr M. Truchan-Graczyk, Dr F. Boyer, Dr M. Dib, Dr C. Foussard, Dr S. Francois, Dr M. Gardembas, Dr F. Genevieve, Dr M. Hunault, Dr A. Schmidt; CH Annecy: Dr F. Orsini-Piocelle, Dr P. Cony-Makhoul, Dr B. Corront, Dr N. Daguindau, Dr A. Parry, Dr C. Reynes; CH Antibes-Juan-les-pins: Dr D. Re; CH V. Dupouy Argenteuil: Dr L. Sutton, Dr A. Aljijakli, Dr P Genet, Dr V. Morel, Dr T. Touahri; CH Avignon: Dr H. Zerazhi; CH Bayonne: Dr C. Araujo, Dr A. Banos, Dr A. Thannberger; CH Beauvais: Dr K. Ghomari, Dr J.-L. Dutel; H Jean Minjoz Besançon: Dr A. Brion, Dr E. Daguidau, Dr E. Deconinck, Dr P. Delaby, Dr J. Fontan, Dr M. Heczko, Dr P. Helias, Dr J. Vuillier; CH Béziers: Dr A. Saad; H Haut Leveque Pessac: Dr M.-S. Dilhuydy, Dr K. Bouabdallah, Dr G. Harvet, Dr T. Leguay, Pr N. Milpied, Dr A. Pigneux, Dr A. Schmitt, Dr R. Tabriza, Dr S. Vigouroux; CH Boulogne-sur-mer: Dr B. Choufi; CHU Brest: Dr H. Saad, Dr C. Berthou, Dr J.-R. Eveillard; C François Baclesse: Pr J.-P. Vilque, Dr C. Fruchart, Dr D. Musafira; H Louis Pasteur Chartres: Dr M. Maigre, Dr L. Abdelkader-Aljassem; CHU Clermont-Ferrand: Pr O. Tournilhac, Pr J.-O. Bay, Dr V. Cacheux, Dr C. Chaleteix, Dr B. De Renzis, Dr R. Guieze, Dr E. Hermet, Dr C. Molucon-Chabrot, Dr J. Fleury; H Civils Colmar: Dr M. Belkad; CHSF Corbeil: Dr C. Salanoubat, Dr S. Haiat, Dr B. Joly, Dr C. Petitdidier; CH Henri Mondor: Dr J. Dupuis; CHU Dijon: Dr E. Ferrant, Dr J.-N. Bastie, Dr D. Caillot, Dr O. Casasnovas, Dr I. Lafon, Dr A. Waultier; CHU Grenoble: Dr L. Molina, Dr C.-E. Bulabois, Pr J.-Y. Cahn, Dr S. Courby, Dr F. Garban, Dr R. Gressin, Dr B. Pegourie, Dr A. Thiebaut; CHD La-Roche-sur-Yon: Dr M. Tiab, Dr T. Chatellier, Dr T. L'haridon, Dr H. Maisonneuve, Dr F. Priou, Dr B. Villemagne; CH Le Mans: Dr N. Denizon, Dr K. Le Du; CHRU Lille: Dr B. Cazin, Dr M. Wemeau; CHU Limoges: Dr D. Bordessoule, Dr M.-P. Gourin, Dr A. Jaccard, Dr S. Moreau, Dr L. Remenieas, Dr M. Touati, Dr P. Turlure; CH Lorient: Dr P. Moreau, Dr M.-F. Le Coz, Dr O. Luycx, Dr M. Niault; C Léon Bérard Lyon: Dr C. Sebban; Institut Paoli-Calmettes Marseille: Dr T. Aurran-Schleinitz; CHU Marseille: Dr R. Costello, Dr G. Sebahoun; CH Meaux: Dr W. Abarah, Dr I. Mahfouz ; H Notre Dame De Bon Secours Metz : Dr I. Guibaud, Dr B. Christian, Dr V. Dorvaux, Dr S; Visanica; CHU Montpellier: Pr G. Cartron, Dr C. Autrand, Dr R. Navarro, Dr G. Olivier,

Dr P. Quittet; CH Mulhouse: Dr B. Drenou, Dr J.-C. Eisenmann, Dr Y. Arkam, Dr M. Ojeda-Uribe; CHU Nantes: Dr B. Mahe, Dr S. Ayari, Dr N. Blin, Dr P. Chevallier, Dr J. Delaunay, Dr V. Dubruille, Dr T. Gasinne, Dr T. Guillaume, Dr S. Le Gouill, Dr P. Moreau, Pr M. Mohty, Dr V. Roland; C Catherine De Sienne Nantes: Dr N. Morineau; C Antoine Lacassagne Nice: Dr D. Re; CHU Nimes: Dr E. Jourdan, Dr P. Bourquard, Dr B. Richard, Dr S. Wickenhauser; CHR Orléans: Dr M. Alexis, Dr M. Schoenwald; H Avicenne Bobigny: Pr F. Cymbalista, Dr V. Levy; H Hotel Dieu Paris: Pr F. Dreyfus, Dr S. park; H La Pitié Salpétrière Paris: Pr V. Leblond; H Saint Antoine Paris: Dr Z. Marjanovis, Dr A. Verhoff; H Saint Louis Paris: Dr J.-M. Zini; CH Perpignan: Dr L. Sanhes, Dr S. Burcheri, Dr A. Karangwa; CHU La Miletrie Poitiers: Dr B. Dreyfus; CH Pontoise: Dr H. Gonzalez, Dr R. Benramdane, Dr L. Fouillard; CHU Reims: Pr A. Delmer; CHU Rennes: Pr T. Lamy, Dr M. Bernard, Dr X. Cahu, Dr C. Dauriac, Dr S. De Guibert, Dr M. Escoffre-Barbe, Dr R. Houot, Dr S. Nimubona; C Henri Becquerel Rouen: Pr S. Lepretre; Institut de Cancérologie de la Loire Saint Priest-en-Jarez: Dr K. Augeul-Meunier, Dr J. Cornillon, Dr D. Guyotat, Dr J. Jaubert, Dr C. Mounier, Dr T. Muron, Dr C. Portois, Dr E. Tavernier; CH Saint Quentin: Dr R. Garidi, Dr S. Legrand ; CHU Toulouse : Dr L. Ysebaert, Dr L. Oberic, Dr C. Recher, Dr G. Laurent; CHU Tours: Dr C. Dartigeas, Dr L. benboubker, Pr P. Colombat, Dr E. Gyan, Dr S. Lissandre, Dr M. Delain; CHU Nancy: Pr P. Feugier; CH Vannes: Dr H. Jardel, Dr P. Godmer.

References

1. Hallek M, Cheson BD, Catovsky D, Caligaris-Cappio F, Dighiero G, Dohner H*, et al.* Guidelines for the diagnosis and treatment of chronic lymphocytic leukemia: a report from the International Workshop on Chronic Lymphocytic Leukemia updating the National Cancer Institute-Working Group 1996 guidelines. *Blood* 2008 Jun 15; **111**(12)**:** 5446-5456.

2. Lepretre S, Letestu R, Dartigeas C, Maisonneuve H, Aurran T, Feugier P*, et al.* Results of a Phase II Randomizing Intensified Rituximab Pre-Phase Followed By Standard FCR Vs Standard FCR in Previously Untreated Patients with Active B-Chronic Lymphocytic Leukemia (B-CLL). CLL2010FMP (for fit medically patients): A Study of the french Cooperative Group on CLL and WM (FCGCLL/MW) and the “Groupe Ouest-Est d’Etudes Des Leucémies Aigües Et Autres Maladies Du sang” (GOELAMS) [Poster]. *56th ASH annual meeting* 2014; **3329**.

3. Iwata Y, Matsushita T, Horikawa M, Dilillo DJ, Yanaba K, Venturi GM*, et al.* Characterization of a rare IL-10-competent B-cell subset in humans that parallels mouse regulatory B10 cells. *Blood* 2011 Jan 13; **117**(2)**:** 530-541.

4. DiLillo DJ, Weinberg JB, Yoshizaki A, Horikawa M, Bryant JM, Iwata Y*, et al.* Chronic lymphocytic leukemia and regulatory B cells share IL-10 competence and immunosuppressive function. *Leukemia* 2013 Jan; **27**(1)**:** 170-182.

5. Ouedraogo DE, Makinson A, Kuster N, Nagot N, Rubbo PA, Bollore K*, et al.* Increased T-cell activation and Th1 cytokine concentrations prior to the diagnosis of B-cell lymphoma in HIV infected patients. *Journal of clinical immunology* 2013 Jan; **33**(1)**:** 22-29.

6. Dall'Ozzo S, Andres C, Bardos P, Watier H, Thibault G. Rapid single-step *FCGR3A* genotyping based on SYBR Green I fluorescence in real-time multiplex allele-specific PCR. *Journal of immunological methods* 2003 Jun 1; **277**(1-2)**:** 185-192.

7. Robin X, Turck N, Hainard A, Tiberti N, Lisacek F, Sanchez JC*, et al.* pROC: an open-source package for R and S+ to analyze and compare ROC curves. *BMC bioinformatics* 2011; **12:** 77.
